# Lung Function Variability in Children and Adolescents With and Without Asthma (LUV Study): Protocol for a Prospective, Nonrandomized, Clinical Trial

**DOI:** 10.2196/20350

**Published:** 2020-08-07

**Authors:** Eirini-Sofia Frima, Ilias Theodorakopoulos, Dimos Gidaris, Nikolaos Karantaglis, Grigorios Chatziparasidis, Panagiotis Plotas, Michael Anthracopoulos, Sotirios Fouzas

**Affiliations:** 1 Pediatric Respiratory Unit University Hospital of Patras Patras Greece; 2 Pediatric Respiratory Research Group University of Patras Patras Greece; 3 Electronics Laboratory Department of Physics University of Patras Patras Greece; 4 University of Nicosia Nicosia Cyprus; 5 Pediatric Pulmonology Unit 3rd Pediatric Department Aristotle University of Thessaloniki Thessaloniki Greece; 6 Department of Primary Ciliary Dyskinesia School of Medicine University of Thessaly Larissa Greece; 7 Department of Public Health School of Medicine University of Patras Patras Greece

**Keywords:** asthma, lung function variability, fluctuation analysis, children, adolescents

## Abstract

**Background:**

Variability analysis of peak expiratory flow (PEF) and forced expiratory volume at 1 second (FEV1) has been used in research to predict exacerbations in adults with asthma. However, there is a paucity of data regarding PEF and FEV1 variability in healthy children and adolescents and those with asthma.

**Objective:**

The objective of this study is the assessment of PEF and FEV1 variability in (1) healthy children and adolescents, to define the normal daily fluctuation of PEF and FEV1 and the parameters that may influence it, and (2) children and adolescents with asthma, to explore the differences from healthy subjects and reveal any specific variability changes prior to exacerbation.

**Methods:**

The study will include 100 healthy children and adolescents aged 6-18 years (assessment of normal PEF and FEV1 variability) and 100 children and adolescents of the same age with diagnosed asthma (assessment of PEF and FEV1 variability in subjects with asthma). PEF and FEV1 measurements will be performed using an ultraportable spirometer (Spirobank Smart; MIR Medical International Research) capable of smartphone connection. Measurements will be performed twice a day between 7 AM and 9 AM and between 7 PM and 9 PM and will be dispatched via email to a central database for a period of 3 months. PEF and FEV1 variability will be assessed by detrended fluctuation and sample entropy analysis, aiming to define the normal pattern (healthy controls) and to detect and quantify any deviations among individuals with asthma. The anticipated duration of the study is 24 months.

**Results:**

The study is funded by the “C. Caratheodory” Programme of the University of Patras, Greece (PN 47014/24.9.2018). It was approved by the Ethics Committee (decision 218/19-03-2019) and the Scientific Board (decision 329/02-04-2019) of the University Hospital of Patras, Greece. Patient recruitment started in January 2020, and as of June 2020, 100 healthy children have been enrolled (74 of them have completed the measurements). The anticipated duration of the study is 24 months. The first part of the study (assessment of lung function variability in healthy children and adolescents) will be completed in August 2020, and the results will be available for publication by October 2020.

**Conclusions:**

Healthy children and adolescents may present normal short- and long-term fluctuations in lung function; the pattern of this variability may be influenced by age, sex, and environmental conditions. Significant lung function variability may also be present in children and adolescents with asthma, but the patterns may differ from those observed in healthy children and adolescents. Such data would improve our understanding regarding the chronobiology of asthma and permit the development of integrated tools for assessing the level of control and risk of future exacerbations.

**Trial Registration:**

ClinicalTrials.gov NCT04163146; https://clinicaltrials.gov/ct2/show/NCT04163146

**International Registered Report Identifier (IRRID):**

DERR1-10.2196/20350

## Introduction

Asthma is the most common chronic disease of childhood and represents an important cause of morbidity worldwide [1]. The disease is characterized by episodes of reversible airway obstruction (exacerbations), with specific symptoms (such as wheezing, dyspnea, coughing, chest tightness) and a decrease in peak expiratory flow (PEF) and forced expiratory volume at 1 second (FEV1) [2]_._ However, lung function changes occur in parallel with clinical deterioration, thus presenting limited ability to predict the exacerbation of the disease [3].

Both PEF and FEV1 demonstrate significant daily variability, in relation to circadian and day-by-day fluctuation of measured values [4,5]. In healthy individuals, the pattern of these fluctuations remains constant over long time periods (weeks or months); this contrasts with patients with asthma, for whom PEF and FEV1 variability increase with loss of disease control, especially prior to exacerbations [5,6]. Thus, lung function variability analysis has been used in research to recognize high-risk patients, predict asthma exacerbations, and evaluate the effectiveness of treatment [6-10].

In clinical practice, however, the evaluation of lung function variability requires daily measurements with portable devices, the recording of PEF and FEV1 values in specialized diaries, and periodic evaluations of the data by the attending physicians [2]. The whole process may be both complicated and time-consuming, reducing patients’ adherence—especially in the cases of children and adolescents [11,12]. In addition, the periodic post hoc review of measurements may hamper the prediction of exacerbations, as the time of evaluation may not coincide with changes in the variability of lung function that characterize the loss of asthma control [12].

In recent years, technological advancements in the field of biosensors and microprocessors have permitted the development of reliable, low-cost, ultraportable spirometers, able to connect with cutting-edge mobile phones (smartphones) and monitor lung function parameters in real time and from a distance [13]. The introduction of such devices in clinical practice may overcome most of the aforementioned barriers in following up lung function parameters in the long term [13].

Currently, there is a paucity of data regarding long-term PEF and FEV1 variability in children and adolescents; this holds particularly true for children and adolescents with asthma. Such data would improve our understanding regarding the chronobiological aspects of the disease and may permit the development of integrated tools for assessing the level of asthma control and the risk of future exacerbations.

## Methods

### Study Objectives and Hypotheses

The first objective of the study (objective 1) is the assessment of lung function variability in healthy children and adolescents, focusing on the range and pattern of short- and long-term fluctuations of PEF and FEV1 and the parameters that may influence them. The second objective (objective 2) is the assessment of lung function variability in children and adolescents with asthma; the pattern of short- and long-term PEF and FEV_1_ variability will be established and the differences from healthy subjects will be defined. Specific changes in the variability pattern prior to exacerbation will also be sought and described (objective 3).

We hypothesize that healthy children and adolescents present normal short- and long-term variability of PEF and FEV1, and that the pattern of variability is influenced by age, sex, and environmental stimuli (season, weather conditions, viral infections, etc). Significant lung function variability is also expected for children and adolescents with asthma; the patterns of variability in children and adolescents with asthma may differ from those observed in healthy children and adolescents, being also influenced by environmental conditions and treatment modalities (controller therapy) in a distinguishable way. Finally, we hypothesize that changes in the pattern of lung function variability occur prior to the loss of asthma control and, thus, may be used to predict the exacerbations of the disease.

### Study Design

The study was designed as a nonrandomized, open-label, interventional clinical trial with single group assignment (2 study groups).

### Study Population

The study will include a cohort of healthy children and adolescents (n=100) for the assessment of normal PEF and FEV1 variability (objective 1) and a cohort of children and adolescents with asthma (n=100) for the assessment of PEF and FEV1 variability in this population (objective 2), as well as an investigation of its potential clinical relevance (objective 3). Participants will be recruited at the Pediatric Respiratory Unit and the Department of Pediatrics of the University Hospital of Patras, and at private pediatric offices in the city of Thessaloniki, Korinth, and Trikala, Greece. Each participant will receive a unique study number (comprising 2 letters and 8 numbers in random order).

### Sample Size Estimation

Data regarding long-term PEF and FEV1 variability in children and adolescents (with or without asthma) are lacking. Based on lung function variability, data from adults with asthma [14], and assuming a maximum dropout rate of 10%, we estimated that a sample size of 200 children and adolescents (100 in each group) would allow us to detect a difference of at least 1% in PEF or FEV1 coefficient of variation (CV) between healthy participants and participants with asthma, with 90% power at the 0.05 level. Sample size estimation was performed using the G*Power software (version 3.1.6) [15] after assuming a nonparametric distribution of lung function parameters (Wilcoxon rank-sum test).

### Inclusion and Exclusion Criteria

Inclusion criteria for both cohorts are individuals 6-18 years old; who have access to (or whose parents have access to) a smartphone with internet connection (Wi-Fi or mobile data); and who have given (or whose parents have given) informed, written consent to participate.

Inclusion criteria for the cohort of healthy children and adolescents are no asthma diagnosis or prescription of relevant medication (beta-2 agonists, anticholinergics, inhaled corticosteroids or montelukast) in the last 2 years; and normal baseline spirometry, defined as FEV1 and FEV1/FVC > 80% of predicted (Global Lung Initiative [GLI] normative data [16]) without significant reversibility (FEV1 change < 10%) after administration of 300 μg salbutamol inhaler.

Inclusion criteria for the cohort of children and adolescents with asthma are doctor-diagnosed mild or moderate asthma [2] in the last 2 years; administration of controller therapy for at least 6 months in the previous year; and at least one spirometry, with FEV1 and FEV1/FVC < 80% of predicted (GLI normative data [16]) in the previous year.

Exclusion criteria for both cohorts are major disabilities (eg, chromosome abnormalities, neurological or muscular disorders, neurodevelopmental delay) that may hamper the proper performance of lung function measurements; respiratory conditions (eg, severe respiratory infection, chest trauma) or other health-related events (eg, surgery, trauma) in the month prior to enrollment or during the 3-month period of observation; failure to complete the run-in period successfully (ie, to perform acceptable spirometries at the predetermined time frames); or inability to perform 3 consecutive measurements or 6 measurements in total (3.3% of the anticipated 180 measurements) within the 3-month period of observation.

### Ethics Approval and Consent to Participate

This study was approved by the Ethics Committee (decision 218/19-03-2019) and the Scientific Board (decision 329/02-04-2019) of the University Hospital of Patras, Greece. The study was registered with ClinicalTrials.gov under the number NCT04163146 (registered on November 14, 2019). Informed consent from parents, or from parents and participants (in the case of adolescents older than 12 years of age), will be obtained at inclusion. The informed consent grants access to the participants’ measurements and medical files, and permits the transmission of data through the internet, given that all conditions of anonymity and data protection are met.

### Lung Function Measurements

PEF and FEV1 measurements will be performed using an FDA-approved ultraportable spirometer (Spirobank Smart; MIR Medical International Research), with a bidirectional digital turbine (flow range ± 16L/s; volume accuracy ±3% or 50 mL; flow accuracy ±5% or 200 mL/s; dynamic resistance <0.5 cm H_2_O/L/s) and capable of connecting to a smartphone via Bluetooth using a dedicated freeware app (iSpirometry, MIR Medical International Research). Apart from PEF and FEV1, the device provides data on the forced expiratory capacity (FVC) and forced expiratory flow between 25% and 75% of FVC (FEF25-75). The app includes graphic incentives to assist in performing technically acceptable tests; it also includes a quality grading system that generates messages to inform whether the measurement was technically correct or, if not, the nature of the mistake (eg, “good blow” for an acceptable test, or “blow faster,” “blow longer,” etc, for nonacceptable maneuvers). Each participant will receive his personal spirometer, which will be paired to one or more smartphones (participant’s device, parents’ device, or both). In a case of two or more participants from the same family, each spirometer will be paired to a separate smartphone. Detailed information regarding use and maintenance of the device will be provided in the form of a printed brochure and online resources available on the study web site [17].

### Protocol

Tests will be performed according to American Thoracic Society/European Respiratory Society (ATS/ERS) standards [18]. The technique will be demonstrated by one of the investigators at enrollment, while detailed information will also be available through the online video resources on the study web site [17]. Measurements will be performed twice a day between 7 AM and 9 AM and between 7 PM and 9 PM. Each participant must perform at least 3 technically acceptable maneuvers. Completed measurements will be dispatched by the participants or their parents to a central database via email (encrypted pdf format).

Eligible children and adolescents will initially be asked to complete a run-in period of 10 days (20 trials) to assess their ability to perform technically acceptable spirometries (daily and at predetermined time frames), dispatch the measurements to the central database, and comply to investigators instructions. Those who will demonstrate satisfactory adherence will proceed to the main study consisting of daily measurements at predetermined time frames for a period of 3 months (90 days, 180 trials).

The expected duration of the study is 24 months and it will include two phases: Phase I and Phase II. Phase I will proceed from January 2020 to September 2020 and will consist of assessments of lung function variability in healthy children and adolescents. Phase II will proceed from October 2020 to December 2021 and will consist of assessments of lung function variability in children and adolescents with asthma.

**Figure 1:**
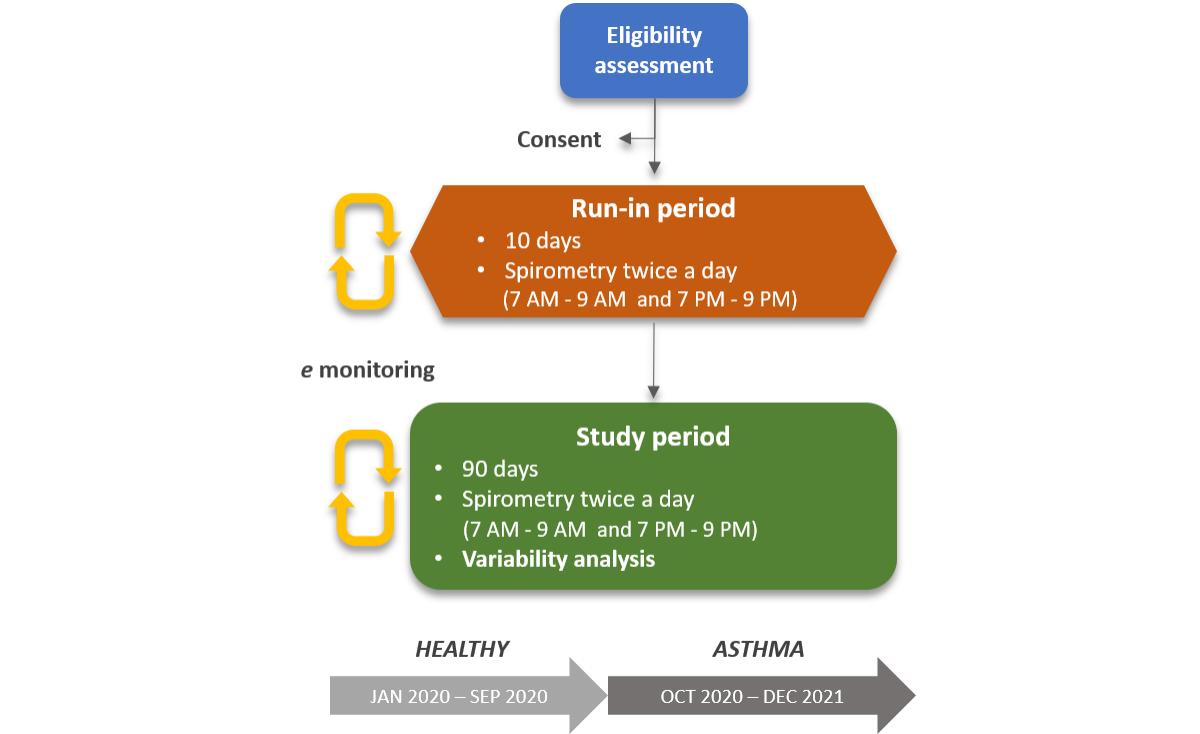
Study flow chart.

**Figure 2:**
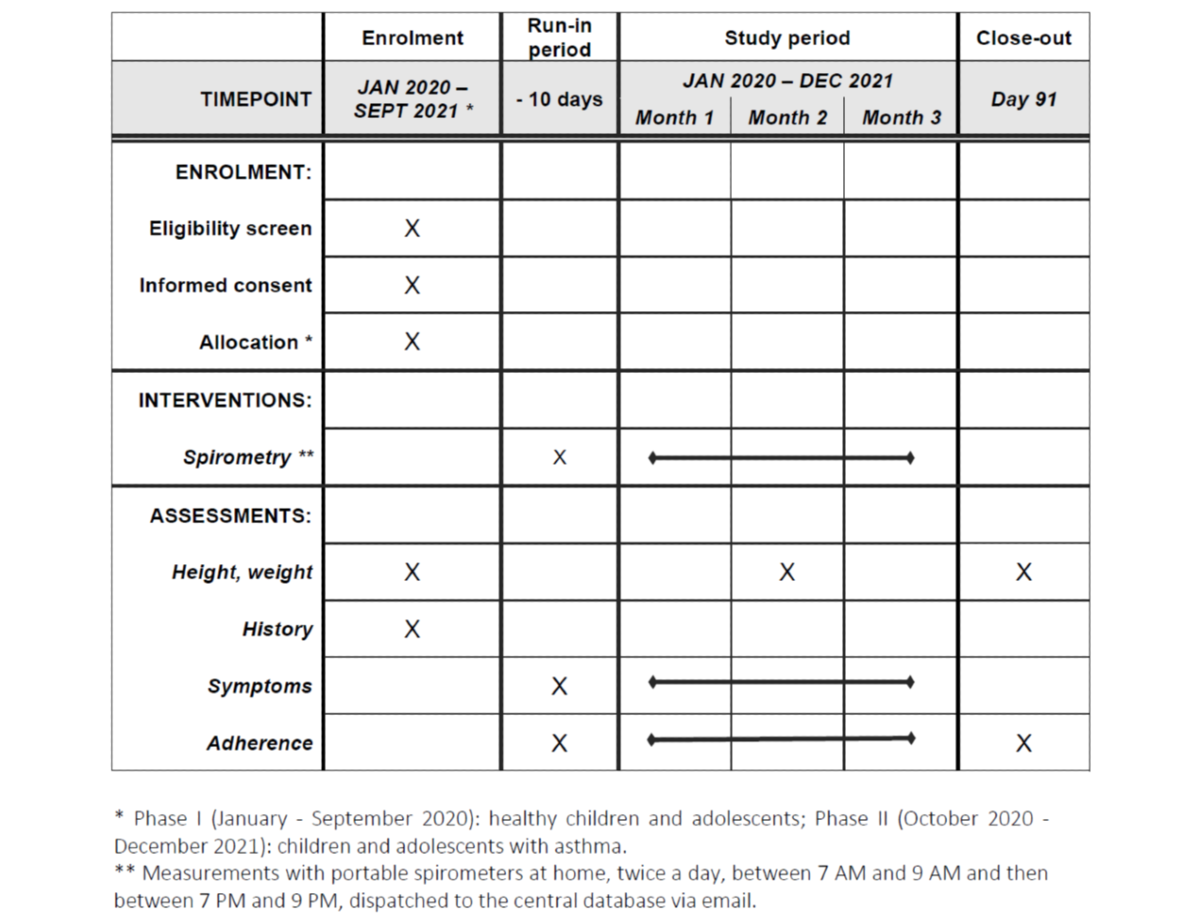
Standard protocol items: recommendations for interventional trials (SPIRIT) checklist.

A flow chart of the study is presented in Figure 1. The protocol is in accordance with SPIRIT (standard protocol items: recommendations for interventional trials) guidelines (Figure 2).

### Data Acquisition and Monitoring

**Figure 3:**
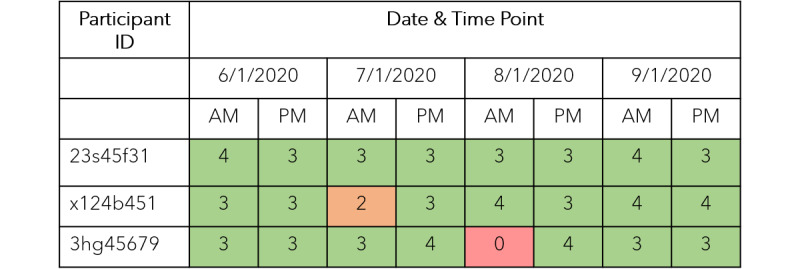
Example of the monitoring table presenting the number of acceptable tests per day/time point for each participant. Green cells: full adherence; orange cells: <3 acceptable measurements; red cells: missed measurements.

PDF files will be downloaded and converted to text files using optical character recognition. PEF, FEV1, FVC, and FEF25-75 values, as well as quality grading of each measurement, will be recognized and introduced in a specific registry. The best PEF, FEV1, FVC, and FEF25-75 values among the technically acceptable trials for a given date and time will be identified and transferred from the registry to a participant-specific file. Additionally, all registry data will be included in a monitoring table that presents the number of acceptable tests per day and time for each participant (Figure 3). PDF downloading and data acquisition will be performed automatically by special scripts implemented in MatLab (version R2019a; MathWorks Inc).

The monitoring table (Figure 3) will be reviewed twice a day (at 11 AM and 11 PM) and participants will be notified by direct telephone contact in case of inappropriate technique or missing measurements.

### Variability Analysis

Participant-specific files will be used for variability analysis, focusing primarily on the variability of PEF and FEV1 and secondarily on FEV1/FVC and FEF25-75. All variables will be transformed into percentage predicted values according to GLI normative data [16].

Lung function variability will be assessed in 3 ways: (1) standard variability indices, (2) detrended fluctuation analysis (DFA), and (3) sample entropy (SampEn).

Standard variability indices such as CV (defined as SD divided by the mean) will be used. To avoid any bias due to the presence of trends within the time series, CV will also be calculated as the average of 24 moving windows (length 14 measurements; step 7 measurements).

DFA is a method that has been widely used for the investigation of intrinsic correlation within time series [19]. Initially, the square root of the time series F(*n*) is calculated for segments of different (time) length *n*. A linear relationship in the logarithmic graph F(*n*) - log(*n*) indicates the existence of fractal architecture in the scaling of the specific data, while the slope *a* of the line describes the pattern of long-term fluctuations [19]. A change in daily variability of PEF or FEV1 results in a simultaneous deviation from the predetermined *a* value [6]. This deviation can easily be detected and quantified. It has been shown that the magnitude of *a* deviation reflects the likelihood of asthma exacerbation within the next month [6].

SampEn is a measure of increased irregularity or complexity, which relies on the identification of recurrent patterns within a nonstationary time series (ie, the probability that a series of points within the signal will repeat themselves at a subsequent time-point) [20]. Within a regular and less complex system, the frequency of sequence matches is high; therefore, the entropy is low. SampEn has emerged as a more reliable index of dynamic variability, mainly because it is relatively independent of the length of the time series [20].

Variability analysis will be performed in the MatLab environment.

### Additional Data

Patients’ characteristics (age, sex, place of residence, baseline lung function, allergies, comorbidities, type of medication, etc) will be recorded. The effect of these parameters on the pattern of lung function variability will also be explored.

### Statistics

Normally distributed data will be presented as means (SD) and compared with Student’s *t* test, while nonparametric data will be presented as medians with ranges and compared with the Mann-Whitney *U* test. A chi-square or Fisher exact test will be applied to compare different frequencies between the study groups. Multivariable linear regression analyses will be used to explore the effect of various parameters on lung function variability. All analyses will be performed with MatLab and SPSS software (version 25.0; IBM Corp).

## Results

The study is funded by the “C. Caratheodory” Programme of the University of Patras, Greece (PN 47014/24.9.2018). It was approved by the Ethics Committee (decision 218/19-03-2019) and the Scientific Board (decision 329/02-04-2019) of the University Hospital of Patras, Greece.

Patient recruitment commenced in January 2020, and the trial is scheduled to end in January 2022. As of June 2020, 100 healthy children have been enrolled (74 of them have completed the measurements). The anticipated duration of the study is 24 months. The first part of the study, the assessment of lung function variability in healthy children and adolescents, will be completed in August 2020, and the results will be available for publication by October 2020.

Access to the study dataset will be limited to the investigators. After the analysis and publication of the results, the study database will be made available by the corresponding author upon reasonable request. Results will be published in peer-reviewed journals and will be presented in relevant conferences. Authorships will follow the Vancouver declaration.

## Discussion

Increased lung function variability correlates with the frequency of respiratory symptoms in the general population [21] and is indicative of poor asthma control in patients with asthma [7-9,14].

Frey et al [6] applied DFA on 300 consecutive PEF values from a cohort of adults with asthma treated in a crossover manner with regular short-acting beta-2 agonists (SABA), regular long-acting beta-2 agonists (LABA), or placebo. They showed that long-term PEF variability was increased in the SABA phase, which meant that there were several periods of decreased lung function (increased vulnerability and high probability of exacerbation). Conversely, PEF variability was decreased during the LABA phase, signifying that lung function remained persistently within normal limits (decreased vulnerability and low probability of exacerbation). Thamrin et al [8] showed that the self-similarity of PEF values over time correlates well with the loss of asthma control within two weeks following the withdrawal of inhaled corticosteroids. Their findings were later corroborated by Kaminsky et al [14] in a large clinical trial of adults with asthma under controller therapy. Thamrin et al [22] have also demonstrated that the probability of asthma exacerbation at the individual level can be estimated by combining the autocorrelation properties of PEF over time with the absolute PEF value at a specific time point, indicating that both are significant for predicting a loss of disease control in the near future.

Regarding SampEn, Veiga et al [23] found that the entropy of airflow time series was reduced in subjects with asthma compared to healthy controls; lower entropy was associated with more severe airflow obstruction (forced oscillations technique) in that study. More recently, Dames et al [24] investigated the entropy of airflow in patients with chronic obstructive pulmonary disease (COPD) and reported that the SampEn of airflow during resting breathing decreased in proportion to the degree of airway obstruction. Similar data in children and adolescents with or without asthma are lacking.

To remain stable yet adaptable to change, any physiological system needs to balance between order and chaos [25]. Asthma appears to be associated with increased order (reduced complexity) and increased variability of lung function, resulting in wide fluctuations that may lead to periods of increased vulnerability and reduced adaptability to a changing environment [26]. From a clinical standpoint, these features translate into more frequent exacerbations and poor control of the disease. Thus, when properly quantified, changes in lung function over time may reflect both past and future control of asthma [26].

Healthy children and adolescents may present normal short- and long-term fluctuations in lung function; the pattern of this variability may be influenced by age, sex, and environmental conditions. Significant lung function variability may also be present in children and adolescents with asthma, but the patterns may differ from those observed in healthy children and adolescents. Such data would improve our understanding regarding the chronobiology of asthma and may permit the development of integrated tools for assessing the level of control and risk of future exacerbations.

## Appendix

Multimedia Appendix 1 Funding Approval.

## Abbreviations

COPD: chronic obstructive pulmonary disease
CV: coefficient of variation
DFA: Detrended Fluctuation Analysis
FEF25-75: forced expiratory flow between 25% and 75% of FVC
FEV1: forced expiratory volume at 1 second
FVC: forced expiratory capacity
GLI: Global Lung Initiative
LABA: long-acting beta-2 agonists
PEF: peak expiratory flowSampEn: Sample entropy
SABA: short-acting beta-2 agonists

## References

[1] (2019). Chronic respiratory diseases: asthma. World Health Organization, Geneva.

[2] Global Strategy for asthma management and prevention, 2019. Global Initiative for Asthma (GINA).

[3] Papi A, Brightling C, Pedersen SE, Reddel HK (2018). Asthma. Lancet.

[4] Spengler CM, Shea SA (2000). Endogenous circadian rhythm of pulmonary function in healthy humans. Am J Respir Crit Care Med.

[5] Martin RJ, Banks-Schlegel S (1998). Chronobiology of asthma. Am J Respir Crit Care Med.

[6] Frey U, Brodbeck T, Majumdar A, Taylor DR, Town GI, Silverman M, Suki B (2005). Risk of severe asthma episodes predicted from fluctuation analysis of airway function. Nature.

[7] Thamrin C, Stern G, Strippoli MF, Kuehni CE, Suki B, Taylor DR, Frey U (2009). Fluctuation analysis of lung function as a predictor of long-term response to beta2-agonists. Eur Respir J.

[8] Thamrin C, Taylor DR, Jones SL, Suki B, Frey U (2010). Variability of lung function predicts loss of asthma control following withdrawal of inhaled corticosteroid treatment. Thorax.

[9] Thamrin C, Nydegger R, Stern G, Chanez P, Wenzel SE, Watt RA, FitzPatrick S, Taylor DR, Frey U (2011). Associations between fluctuations in lung function and asthma control in two populations with differing asthma severity. Thorax.

[10] Delgado-Eckert E, Fuchs O, Kumar N, Pekkanen J, Dalphin J, Riedler J, Lauener R, Kabesch M, Kupczyk M, Dahlen S, Mutius EV, Frey U, PASTUREBIOAIR Study groups (2018). Functional phenotypes determined by fluctuation-based clustering of lung function measurements in healthy and asthmatic cohort participants. Thorax.

[11] Ginsberg D (2009). An unidentified monster in the bed--assessing nocturnal asthma in children. Mcgill J Med.

[12] Kamps AW, Roorda RJ, Brand PL (2001). Peak flow diaries in childhood asthma are unreliable. Thorax.

[13] Aliverti A (2017). Wearable technology: role in respiratory health and disease. Breathe (Sheff).

[14] Kaminsky DA, Wang LL, Bates JHT, Thamrin C, Shade DM, Dixon AE, Wise RA, Peters S, Irvin CG (2017). Fluctuation Analysis of Peak Expiratory Flow and Its Association with Treatment Failure in Asthma. Am J Respir Crit Care Med.

[15] Faul F, Erdfelder E, Lang A, Buchner A (2007). G*Power 3: a flexible statistical power analysis program for the social, behavioral, and biomedical sciences. Behav Res Methods.

[16] Quanjer PH, Stanojevic S, Cole TJ, Baur X, Hall GL, Culver BH, Enright PL, Hankinson JL, Ip MSM, Zheng J, Stocks J, ERS Global Lung Function Initiative (2012). Multi-ethnic reference values for spirometry for the 3-95-yr age range: the global lung function 2012 equations. Eur Respir J.

[17] Lung Function Variability in Children and Adolescents Study.

[18] Graham BL, Steenbruggen I, Miller MR, Barjaktarevic IZ, Cooper BG, Hall GL, Hallstrand TS, Kaminsky DA, McCarthy K, McCormack MC, Oropez CE, Rosenfeld M, Stanojevic S, Swanney MP, Thompson BR (2019). Standardization of Spirometry 2019 Update. An Official American Thoracic Society and European Respiratory Society Technical Statement. Am J Respir Crit Care Med.

[19] Peng C- K, Mietus J, Hausdorff JM, Havlin S, Stanley HE, Goldberger AL (1993). Long-range anticorrelations and non-Gaussian behavior of the heartbeat. Phys Rev Lett.

[20] Gonem S, Umar I, Burke D, Desai D, Corkill S, Owers-Bradley J, Brightling CE, Siddiqui S (2012). Airway impedance entropy and exacerbations in severe asthma. Eur Respir J.

[21] Boezen HM, Schouten JP, Postma DS, Rijcken B (1995). Relation between respiratory symptoms, pulmonary function and peak flow variability in adults. Thorax.

[22] Thamrin C, Zindel J, Nydegger R, Reddel HK, Chanez P, Wenzel SE, FitzPatrick S, Watt RA, Suki B, Frey U (2011). Predicting future risk of asthma exacerbations using individual conditional probabilities. J Allergy Clin Immunol.

[23] Veiga J, Lopes AJ, Jansen JM, Melo PL (2011). Airflow pattern complexity and airway obstruction in asthma. J Appl Physiol (1985).

[24] Dames KK, Lopes AJ, de Melo PL (2014). Airflow pattern complexity during resting breathing in patients with COPD: effect of airway obstruction. Respir Physiol Neurobiol.

[25] Macklem PT (2008). Emergent phenomena and the secrets of life. J Appl Physiol (1985).

[26] Kaminsky DA, Irvin CG (2015). What long-term changes in lung function can tell us about asthma control. Curr Allergy Asthma Rep.

