# Supplementary material for: Lung Function Variability in Children and Adolescents With and Without Asthma (LUV Study): Protocol for a Prospective, Nonrandomized, Clinical Trial
Source: JMIR Res Protoc. 2020 Aug 7;9(8):e20350. doi: 10.2196/20350 (PMC7442949; doi:10.2196/20350)
Supplement: Multimedia Appendix 1 [file resprot_v9i8e20350_app1.pdf]

HELLENIC REPUBLIC

(LOGOTYPE OF THE UNIVERSITY OF PATRAS)

SPECIAL ACCOUNT FOR RESEARCH FUNDS
P.D. 432/81
http://research.upatras.gr
Info: Fr. Polyzogopoulou & St. Mathiou
Tel: 2610/997947 & 969056
E-mail: researchsup@upatras.gr

Patras, 24/9/2018
PN: 47014

SUBJECT: "C. Caratheodori" Programme- Results of invitation 2017

Dear colleagues,

We hereby inform you that the evaluation of proposals submitted in the context of the call "C. Caratheodori 2017" has been concluded.

Following the online submission of the proposals at the platform of "C.CARATHEODORI" programme, each proposal was assigned to two external reviewers -experts in the respective scientific field - for evaluating the overall quality and the particulars, the scientific International impact and the methodological integrity of the proposed project, as well as the competence and the CV of the scientific supervisor. Each reviewer received a personal code to access the online platform which included the assigned proposal(s) and the rating tools.

Subsequently, field-specific Review Committees were organized and met at independent locations (Piraeus Bank Historical Archive in Athens & National Technical University of Athens) to jointly review the proposals and finalize their recommendations. It should be emphasized that all reviewers who participated even the ones who were present via teleconference, praised the evaluation process as a whole. In particular, the organization of meetings of the University's Departments, outside the facilities of the University of Patras and without the presence of the Steering Committee of "C. Caratheodori" programme, was recognized as a critical measure to ensure the absolute independence of the reviewers and lead the Review Committees to unbiased decisions.

According to the original call a maximum of 11 proposals would be funded, in relation with the number of the proposals submitted by University's Departments, taking into account their quality. However, the Research Committee of the University of Patras, based on the recommendation of the Steering Committee of "C. Caratheodori" programme, preapproved the funding of the 3 proposals from the School of Engineering, 2 from the School of Humanities and Social Sciences, 3 from the School of Natural Sciences, 1 from the School of Economics & Business and 3 proposals from the School of Health Sciences.

Therefore the 12 proposals that are eligible for funding by 30.000 € each for the period 2019-2021 are the following:
[Names of Scientific Supervisors were masked for personal data protection]

Call C. Caratheodori 2017: Proposals eligible for funding

School of Engineering

No	Scientific Supervisor	Department	Title
1	[redacted]	Chemical Engineering	Bimetallic catalysts for the selective transformation of biomass molecules to high value-added chemicals
2	[redacted]	Mechanical and Aeronautical Engineering	Theoretical, numerical and experimental investigation of the fracture mechanics of the delamination fatigue of adhesively bonded joints (TALENTED)
3	[redacted]	Chemical Engineering	Structure and function of novel oxidases implicated in biomass degradation

School of Humanities and Social Sciences

No	Scientific Supervisor	Department	Title
1	[redacted]	Education Sciences	Intercultural capital and teacher's competence
2	[redacted]	Literature	The history of a motion verb

School of Health Sciences

No	Scientific Supervisor	Department	Title
1	[redacted]	Medicine	Investigation of the role of polymorphisms in the function and mechanism of vitamin D receptor (VDR) gene regulation in patients with cirrhosis.
2	Fouzas Sotirios	Medicine	Lung function variability in children and adolescents for predicting asthma exacerbations
3	[redacted]	Medicine	Platelet REactivity among Patients on Anti-Retroviral Therapy (PREP-ART)

School of Economics & Business

No	Scientific Supervisor	Department	Title
1	[redacted]	Management of Cultural Environment and New Technologies	Digital and public history: intersecting theories and methodologies of historical studies and new technologies

School of Natural Sciences

No	Scientific Supervisor	Department	Title
1	[redacted]	Chemistry	Novel matrix-based approaches in breast cancer therapy: Hyaluronan synthesis inhibition by 4-MU and salicylate
2	[redacted]	Chemistry	"Smart" Nanoparticles Suitable as Thermometers and Fluorescence Detectors in Magnetic Resonance Imaging
3	[redacted]	Τμήμα Επιστήμης των Υλικών	Electrochemical based Sensors On Flexible Substrates (ESOFS)

Scientific Supervisors may access their proposal, as well as the reviewers' comments and rating, through the online platform. Specifically, Scientific Supervisors must login to the platform using their personal credentials and then follow the special link.

As already mentioned, after the initial evaluation the field-specific Review Committee met to jointly review the proposals and finalize its recommendations to avoid possible mistakes. As a result, an objection procedure is not provided, except for technical issues.

The implementation of the proposed projects may begin immediately after the receipt of this letter and not later than December 1, 2018.

I would like to thank all Scientific Supervisors for participating in the call "C. Caratheodori 2017" and for the high quality of the submitted proposals. According to the Reviewers' committee all the 36 proposals submitted were distinguished for their quality.
More information about "C. Caratheodori" funding programme can be found on the website.

On behalf of the Steering Committee of the funding program "C. Caratheodori".

Professor Demosthenes K. Polyzos
(Signature – University Stamp)

Chairman of the Research Committee of the University of Patras

Deputy Rector of Research & Development

I the undersigned attorney at law duly authorized by law confirm that this is the true and exact translation of the attached herewith document from Greek Language into English language. Also the undersigned attorney at law has the scientific knowledge to translate the above document.

Patras, 10-1-2020

THE TRANSLATOR ATTORNEY AT LAW

DIAMANTOPOULOU ELENI - MARIA
ATTORNEY AT LAW
PATRAS BAR No.: 1572
28-30 KANARI STR. GR-262 22 PATRAS
TEL: +302610321444 - FAX: +302610321724
V.A.T. No.: 119944192
TAX OFFICE: 3rd PATRAS

ΕΛΛΗΝΙΚΗ ΔΗΜΟΚΡΑΤΙΑ
ΔΙΚΗΓΟΡΙΚΟΣ ΣΥΛΛΟΓΟΣ ΠΑΤΡΩΝ
It is certified that this is the true signature of the attorney at law of Patras Bar Association
DIAMANTOPOULOU ELENI-MARIA
Patras Greece 10/1/2020
The President of Patras Bar Association
Athanasios Zoupas

Ε Λ Λ Η Ν Ι Κ Η   Δ Η Μ Ο Κ Ρ Α Τ Ι Α

ULOU ELENI-MARIA
KNEY AT LAW
AS BAR No.: 1572
RI STR. GR-262 22 PATRAS
21444 - FAX: +302610321724
No.: 119944192
FICE: 3rd PATRAS

ΕΙΔΙΚΟΣ ΛΟΓΑΡΙΑΣΜΟΣ ΚΟΝΔΥΛΙΩΝ ΕΡΕΥΝΑΣ
Π. Δ. 432/81
http://research.upatras.gr
Πληροφορίες: Φρ. Πολυζογωπούλου & Στ. Μαθιού
ΤΗΛ: 2610/ 997947 & 96 9056
E-mail: researchsup@upatras.gr

Πάτρα, 24-9-2018
Αρ. Πρωτ.: 47014

ΘΕΜΑ: *Πρόγραμμα «Κ. Καραθεοδωρή» - Αποτελέσματα πρόσκλησης 2017*

Αγαπητοί Συνάδελφοι,

Με την παρούσα σας ενημερώνουμε ότι η διαδικασία κρίσης των προτάσεων που υποβλήθηκαν στο πλαίσιο της πρόσκλησης «Κ. Καραθεοδωρή 2017» ολοκληρώθηκε.

Κατόπιν της ηλεκτρονικής υποβολής των προτάσεων στη πλατφόρμα του προγράμματος «Κ. ΚΑΡΑΘΕΟΔΩΡΗ», κάθε πρόταση δόθηκε προς κρίση σε δύο κριτές, ειδικούς στο εκάστοτε επιστημονικό πεδίο ώστε να αξιολογήσουν, συνολικά την ποιότητα της πρότασης αλλά και τα ακόλουθα επιμέρους στοιχεία, ήτοι την Διεθνή διάσταση και ανταγωνιστικότητα της πρότασης, την Μεθοδολογική επάρκεια και ικανότητα διεκπεραίωσης και το βιογραφικό του Επιστημονικά Υπεύθυνου. Σε κάθε κριτή δόθηκε προσωπικός κωδικός για την ηλεκτρονική πλατφόρμα του προγράμματος, δίνοντας πρόσβαση στα συγκεκριμένα ηλεκτρονικά αρχεία των προτάσεων ευθύνης του, όπως και στο πεδίο ηλεκτρονικής καταχώρησης βαθμολογίας και σχολίων.

Μετά την ολοκλήρωση της κρίσης, πραγματοποιήθηκαν Επιτροπές Κριτών, που έλαβαν χώρα στο Ιστορικό Αρχείο της Τράπεζας Πειραιώς στην Αθήνα και στο Εθνικό Μετσόβιο Πολυτεχνείο. Θα ήθελα να τονίσω ότι, οι κριτές, τόσο οι παρόντες δια ζώσης, όσο και οι παρόντες μέσω τηλεδιάσκεψης, εξήραν την διαδικασία αξιολόγησης στο σύνολό της. Ιδιαίτερα, η οργάνωση των συνεδριάσεων ανά Σχολή σε χώρο εκτός του Πανεπιστημίου και χωρίς την παρουσία των μελών της Επιτροπής του Προγράμματος, υπογράμμισε την μέριμνα της Επιτροπής για ανεξάρτητη και ανεπηρέαστη κρίση.

Σύμφωνα με την προκήρυξη, προβλεπόταν η χρηματοδότηση 11 συνολικά προτάσεων, αναλογικά με το πλήθος των προτάσεων που υποβλήθηκαν ανά Σχολή, λαμβάνοντας υπόψη πάντα την ποιότητά τους. Σύμφωνα με τα παραπάνω, η Επιτροπή Ερευνών, κατόπιν εισήγησης της Επιτροπής του προγράμματος «Κ. Καραθεοδωρή» προέκρινε την χρηματοδότηση των επικρατέστερων 3 προτάσεων από την Πολυτεχνική Σχολή, 2 προτάσεων από την Σχολή Ανθρωπιστικών & Κοινωνικών Επιστημών, 3 προτάσεων από την Σχολή Θετικών Επιστημών, 1 πρότασης από την Σχολή Οργάνωσης & Διοίκησης Επιχειρήσεων και 3 προτάσεων από την Σχολή Επιστημών Υγείας.

OU... ENI - MARIA
AT LAW
R No.: 1572
GR-262 22 PATRAS
FAX. +302610321724
119944192
3rd PATRAS

Ως εκ τούτου οι 12 προτάσεις που προκρίνονται προς χρηματοδότηση είναι οι κάτωθι:

ΧΡΗΜΑΤΟΔΟΤΟΥΜΕΝΑ ΕΡΓΑ "Κ. ΚΑΡΑΘΕΟΔΩΡΗ 2017-ΒΑΣΙΚΗ ΕΡΕΥΝΑ"

Πολυτεχνική Σχολή

Α/Α	Ονοματεπώνυμο Επιστημονικού Υπευθύνου	Τμήμα	Τίτλος
1	[redacted]	Τμήμα Χημικών Μηχανικών	Bimetallic catalysts for the selective transformation of biomass molecules to high value-added chemicals
2	[redacted]	Τμήμα Μηχανολόγων και Αεροναυπηγών Μηχανικών	Theoretical, numerical and experimental investigation of the fracture mechanics of the delamination fatigue of adhesively bonded joints (TALENTED)
3	[redacted]	Τμήμα Χημικών Μηχανικών	Structure and function of novel oxidases implicated in biomass degradation

Σχολή Ανθρωπιστικών Επιστημών

Α/Α	Ονοματεπώνυμο Επιστημονικού Υπευθύνου	Τμήμα	Τίτλος
1	[redacted]	Τμήμα Επιστημών της Εκπαίδευσης και της Αγωγής στην Προσχολική Ηλικία	Διαπολιτισμικό Κεφάλαιο και Ικανότητα Εκπαιδευτικών
2	[redacted]	Τμήμα Φιλολογίας	Ας πα να πηαίνω...Πολυγραμματικοποιήσεις στην ιστορία ενός ρήματος κίνησης

Σχολή Επιστημών Υγείας

Α/Α	Ονοματεπώνυμο Επιστημονικού Υπευθύνου	Τμήμα	Τίτλος
1	[redacted]	Τμήμα Ιατρικής	Διερεύνηση του ρόλου των πολυμορφισμών στη λειτουργικότητα και στο μηχανισμό ρύθμισης του γονιδίου του υποδοχέα της βιταμίνης D (VDR) σε ασθενείς με κίρρωση του ήπατος.
2	Φούζας Σωτήριος	Τμήμα Ιατρικής	Ανάπτυξη συστήματος πρόβλεψης ασθματικών παροξυσμών σε παιδιά και εφήβους μέσω ανάλυσης της διακύμανσης της αναπνευστικής λειτουργίας
3	[redacted]	Τμήμα Ιατρικής	Platelet REactivity among Patients on Anti-Retroviral Therapy (PREP-ART)

Σχολή Οργάνωσης και Διοίκησης Επιχειρήσεων

Α/Α	Ονοματεπώνυμο Επιστημονικού Υπευθύνου	Τμήμα	Τίτλος
1	[redacted]	Τμήμα Διαχείρισης Πολιτισμικού Περιβάλλοντος και Νέων Τεχνολογιών	Ψηφιακή και δημόσια ιστορία: διασταυρούμενες θεωρίες και μεθοδολογίες των ιστορικών σπουδών και των νέων τεχνολογιών

Σχολή Θετικών Επιστημών			
Α/Α	Ονοματεπώνυμο Επιστημονικού Υπευθύνου	Τμήμα	Τίτλος
1	[redacted]	Τμήμα Χημείας	Novel matrix-based approaches in breast cancer therapy: Hyaluronan synthesis inhibition by 4-MU and salicylate
2	[redacted]	Τμήμα Χημείας	"Έξυπνα" Νανοσωματίδια Κατάλληλα ως Θερμόμετρα και Ιχνηθέτες Φθορισμού Στη Μαγνητική Απεικονιστική Τομογραφία
3	[redacted]	Τμήμα Επιστήμης των Υλικών	Electrochemical based Sensors On Flexible Substrates (ESOFS)

Σε όλους τους υποβάλλοντες δίνεται η δυνατότητα πρόσβασης στην πρότασή τους, στα σχόλια των κριτών και την βαθμολόγησή τους μέσω της πλατφόρμας του προγράμματος. Πιο συγκεκριμένα, για να δείτε την αξιολόγηση της πρότασής σας, θα πρέπει πρώτα να συνδεθείτε στην πλατφόρμα με τους κωδικούς που υποβάλλατε την πρότασή σας και στη συνέχεια να ακολουθήσετε τον παρακάτω σύνδεσμο.

Όπως αναφέρθηκε και παραπάνω, μετά την βαθμολόγηση οι κριτές συνεδρίασαν ώστε από κοινού να επανελέγξουν και οριστικοποιήσουν την βαθμολογία τους, για την αποφυγή τυχόν σφαλμάτων. Ως εκ τούτου δεν προβλέπεται διαδικασία υποβολής ενστάσεων παρά μόνο για τεχνικά λάθη.

Η έναρξη υλοποίησης των προγραμμάτων μπορεί να αρχίσει αμέσως μετά την παρούσα επιστολή και όχι αργότερα από την *1η Δεκεμβρίου 2018*.

Στο σημείο αυτό θα ήθελα να ευχαριστήσω τον κάθε ένα από εσάς, τόσο για την συμμετοχή σας στην πρόσκληση του προγράμματος «Κ. Καραθεοδωρή 2017», όσο και για την υψηλή ποιότητα των προτάσεων που υποβλήθηκαν. Σύμφωνα με την γενική ομολογία των κριτών και οι 36 υποβληθείσες προτάσεις διακρίθηκαν για την ποιότητά τους σε όλα τα τεθέντα κριτήρια.

Περισσότερες πληροφορίες για το πρόγραμμα «Κ. ΚΑΡΑΘΕΟΔΩΡΗ» μπορείτε να δείτε στον ιστότοπο του προγράμματος.

Εκ μέρους της Επιτροπής του προγράμματος «Κ. Καραθεοδωρή»

ΠΑΝΕΠΙΣΤΗΜΙΟ ΠΑΤΡΩΝ UNIVERSITY OF PATRAS

Καθηγητής Δημοσθένης Κ. Πολύζος,
Πρόεδρος της Επιτροπής Ερευνών του Πανεπιστημίου Πατρών
Αναπληρωτής Πρυτάνεως Έρευνας & Ανάπτυξης
